# Calculation of the Frailty Index and Precautions for Elderly Patients Undergoing Gastrointestinal Cancer Surgery

**DOI:** 10.7759/cureus.78097

**Published:** 2025-01-27

**Authors:** Enes Sahin, Kazim Sahin, Mehmet Esref Ulutas, Yakup Turan, Sertac Ata Güler, Turgay Simsek, Nuh Zafer Cantürk

**Affiliations:** 1 Department of General Surgery, Kocaeli University Faculty of Medicine, Kocaeli, TUR; 2 Department of General Surgery, Kandıra State Hospital, Kocaeli, TUR; 3 Department of General Surgery, University of Health Sciences, Gaziantep City Hospital, Gaziantep, TUR

**Keywords:** cancer colon, co morbidity, frail elderly, frailty index, gastrointestinal neoplasms

## Abstract

Introduction

It is estimated that the world population is getting older. Accordingly, the incidence of GI tract malignancies is increasing in patients over 65 years of age. This study aims to investigate the impact of frailty on the postoperative course of elderly patients with gastrointestinal malignancies (GIM).

Methodology

This study recruited 120 elderly patients who had been operated on for GIM. Variables such as age, readmission rate, duration of hospitalization, and frailty index (FI) criteria (fatigue, endurance, walking/running speed, comorbidities, and weight loss) were determined, and the FI was calculated. The relationship between the FI and postoperative complications in the study participants was then evaluated.

Results

The study involved 120 patients with gastric, colon, and rectal cancers. The mean age of the participants was 72.79 ± 5.4 years, of which 56.6% (68) were male and 43.3% (52) were female. The average hospitalization duration was 6.7 days, and the average ICU stay duration was 2.2 days. There was a correlation between the age of the patients and their FI scores (FIS).

Conclusion

It was determined that the FIS increases in parallel with increasing age among the elderly. Furthermore, the presence of preoperative comorbidities with old age increases postoperative mortality and morbidity.

## Introduction

Gastrointestinal malignancies (GIM) are among the most common malignancies worldwide. Surgical resection, chemotherapy, and radiotherapy are the most important treatment parameters, especially in early stages. GIM are seen in all age groups. However, when it occurs in the elderly, it often leads to poor living conditions, sarcopenia, and malnutrition. It is estimated that the world population is getting older. Accordingly, the incidence of malignancies in this system is increasing in patients over 65 years of age [1].

The proportion of the elderly population in Turkey is growing every year. The population aged 65 and older, which is considered the elderly population, grew by 24.0% over the last five years, from 6 million 895 thousand in 2017 to 8 million 451 thousand in 2023. However, this population increased from 8.3% in 2016 to 9.7% in 2021. The male-female ratio of the elderly population in 2021 was 6:4. According to population projections, the proportion of Turkey’s elderly population is expected to be 11.0% by 2025, 12.9% by 2030, 16.3% by 2040, 22.6% by 2060, and 25.6% by 2080 [1].

Aging and old age are complex phenomena. Biologically, aging is the accumulation of a series of damages occurring at the molecular and cellular levels. This accumulated damage leads to a decrease in physiological capacity over the years, along with an increase in the risk of various diseases, resulting in a decrease in the capacity of the individual. However, the relationship between age and these changes is very weak, as they do not increase linearly with age. Various environmental factors and individual characteristics may affect aging [2].

Frailty is a measure of decreased physiological capacity resulting from deterioration in organ systems and can be distinguished from the aging process and comorbidities [3]. Elderly individuals are at a high risk for adverse events as a consequence of processes that put them under intense stress (e.g., surgery). The elderly are more likely to develop surgical complications, delayed wound healing, and impaired functional status. Frailty is also linked to a higher risk of mortality.

Frailty is believed to be a chronic, progressive condition manifesting as a spectrum; moderately frail individuals may respond to strategies or interventions to improve their clinical manifestations, while severely frail individuals may experience an irreversible premortem state with limited life expectancy [4]. Frailty is an independent factor that negatively impacts postoperative outcomes.

In this study, we aim to establish the predictive capacity of the frailty index (FI) by examining the relationship between the FI and patients’ readmission rate, duration of stay in the intensive care unit, wound site infection, hemoglobin levels, serum albumin levels, weight loss, and conditional status after surgery for GIM. We believe that this study will lead to the development of preoperative assessment protocols or guidelines to manage frailty in patients undergoing surgery for GIM. In addition, we aim to create a standard by analyzing the conditions that patients encounter in the postoperative processes and the treatment methods to be followed.

## Materials and methods

This study was conducted at the Department of General Surgery, Kocaeli University Faculty of Medicine. Before starting the study, approval from the Non-invasive Clinical Research Ethics Committee of Kocaeli University Faculty of Medicine was obtained (09.10.2023. GOKAEK-2023/16.13), detailed information was provided to the participants, and their written informed consent was obtained. The study was conducted in accordance with the ethical guidelines and the Declaration of Helsinki.

Inclusion criteria

Patients aged 65 years and older who underwent surgery for GIM involving the colon, rectum, and stomach.

Exclusion criteria

Patients under 65 years of age, those with inoperable GIM (colon, rectum, and stomach), and patients with missing data.

Between January 1, 2022, and October 1, 2023, the frailty indices of patients over 65 years of age with GIM who underwent surgery were retrospectively analyzed. All patients over 65 years of age who underwent surgery for GIM during the specified dates were included in the study. A total of 120 eligible patients were included in this study. Patient data were collected and the frailty index scores (FIS) of the participants were evaluated by the statistics department. Clinicians asked patients whether they could climb 10 flights of stairs on their own, whether they could walk 200 meters on their own, what chronic diseases they had, whether they had lost weight in the last year, and if so, by what percentage, and recorded all this information. The FISs of the patients were calculated based on the answers to these questions (Table 1). The hospital records of these patients were examined and parameters such as preoperative albumin and hemoglobin values, postoperative wound infection, anastomotic leakage, duration of intensive care unit stay, total duration of hospital stay, and whether there was readmission within 30 days, and if so, the duration of readmission were recorded. These parameters were analyzed according to the FI.

**Table 1:** Detailing the parameters of frailty index.

Parameter	Question	Yes (1)	No (0)
Tiredness	How many of the last four weeks have you felt tired?	In all or most of them	Little or none
Durability	Do you have difficulty climbing 10 flights of stairs alone?	Yes	No
Walking-running	Do you have difficulty walking 200 meters alone?	Yes	No
Disease	Do you have any of these diseases (list exceeds 5 out of 11): hypertension, diabetes mellitus, myocardial infarction, congestive heart failure, angina, asthma, arthritis, stroke, kidney disease, cancer?	Yes	No
Weight loss	Have you lost 5% of your body weight in the last year?	Yes	No
Conclusion	0: Normal 1-2: Prefrail 3-5: Frail		

Statistical analysis

All statistical analyses were performed using IBM SPSS for Windows, version 20.0 (IBM Corp., Armonk, NY, USA). The Shapiro-Wilk test was used to assess the normality of the data distributions. Continuous variables were expressed as the mean ± SD and median (25th-75th percentiles), and categorical variables were expressed as counts (percentages).

Prior to further analyses, both Kolmogorov-Smirnov and Shapiro-Wilk tests were used to assess the normality of the variables. Non-parametric methods were applied for non-normally distributed variables. Between-group comparisons of categorical variables were performed using the Monte Carlo chi-square test. The relationships between numerical variables were evaluated using Spearman’s correlation analysis. A two-sided p-value of <0.05 was considered statistically significant.

## Results

Demographic data

**Figure 1:**
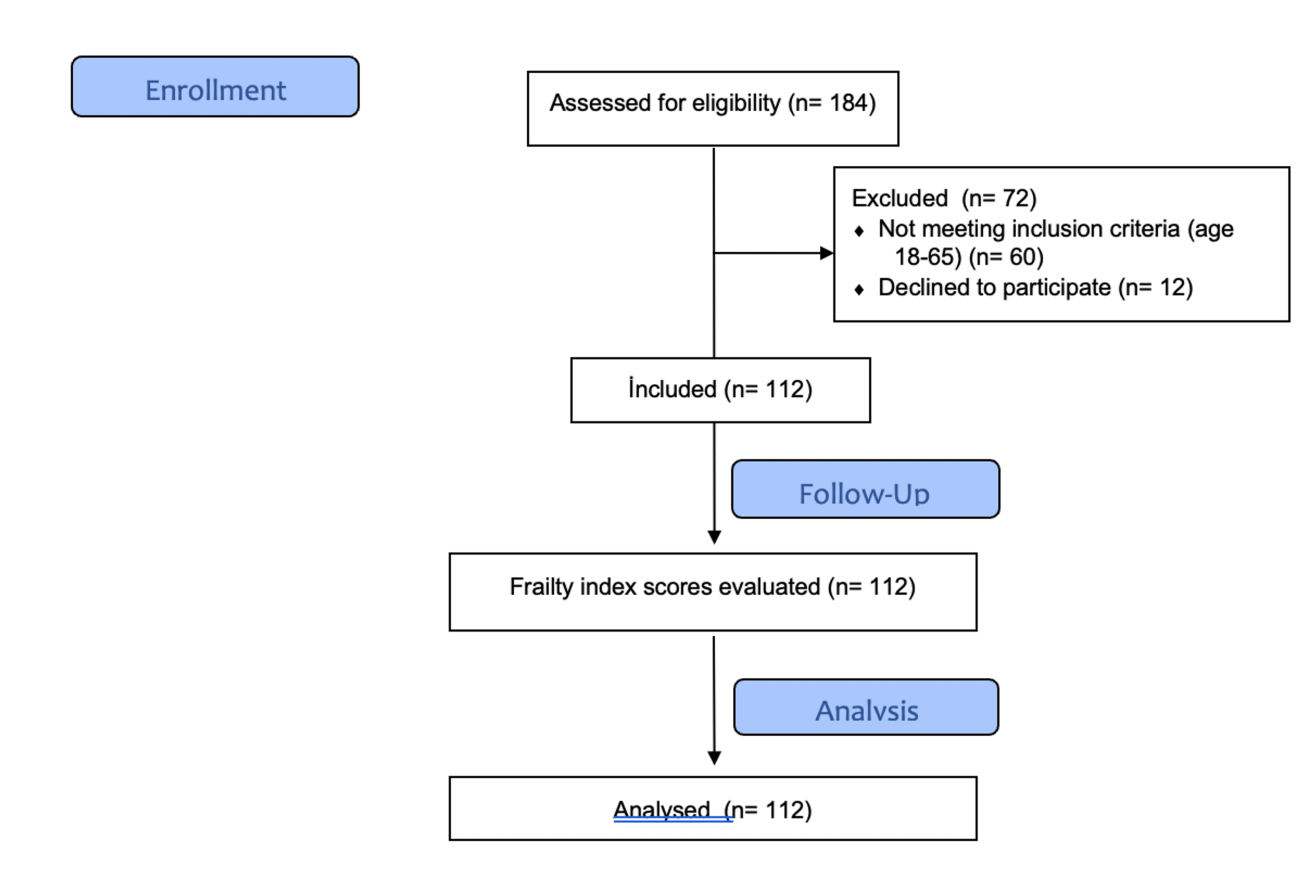
Flow diagram.

The study involved 120 elderly patients who had undergone surgery for colon, rectum, and stomach malignancies (Figure 1). The mean age of the study participants was 72.79 ± 5.443 years; 56.6% (n=68) were male, and 43.3% (n=52) were female.

Classifications of FI

Based on the FI applied to the 120 participants, 19 (15.8%) scored zero and were classified as normal; 49 (40.8%) scored 1-2 and were classified as pre-frail; and 52 (43.3%) scored 3-5 and were classified as frail.

Among the participants with normal FIS, 57.8% (n=11) were male, and 42.2% (n=8) were female. In the pre-frail group, 46.9% (n=23) were male and 53.06% (n=26) were female. In the frail group, 65.3% (n=34) were male and 34.6% (n=18) were female. The demographic data showed no statistically significant relationships between the three subgroups, as determined using the Monte Carlo chi-square test (p = 0.10).

FI and other parameters

The relationship between age and FIS was statistically significant (p = 0.03). The mean duration of hospitalization was 6.74 days, and the mean ICU stay duration was 2.2 days. Hospitalization duration was statistically significantly correlated with FIS (p = 0.048), but not with ICU stay duration (p = 0.90). Age was not statistically significantly correlated with the length of hospitalization or ICU stay duration.

The mean hemoglobin level was 11.15 ± 1.6. Although no statistically significant relationship was found between FI scores and mean hemoglobin levels (p = 0.44), there was a statistically significant relationship between age and mean hemoglobin levels (p = 0.049), with age being negatively correlated with hemoglobin levels.

The mean albumin level was 3.42 ± 0.68. Mean albumin levels had a statistically significant negative correlation with FI scores (p = 0.002) and age (p = 0.006).

Of the 120 participants, 51 had colon malignancy, 27 had gastric malignancy, and 42 had rectal malignancies. Among the gastric malignancy patients, one patient was in the normal frailty group, nine were in the pre-frail group, and 17 were in the frail group. Among the colon malignancy patients, seven were in the normal frailty group, 23 were in the pre-frail group, and 21 were in the frail group. Among the rectal malignancy patients, 11 were in the normal frailty group, 17 were in the pre-frail group, and 14 were in the frail group. When all groups were compared based on a single diagnosis, the relationship between the groups was statistically significant (p = 0.049) (Table 2).

**Table 2:** Classification and comparison of patient groups by cancer type.

	Normal group	Pre-fragile group	Fragile group	Total number of patients	P-value
Colon malignancies	7	23	21	51	<0.001
Gastric malignancies	1	9	17	27	<0.001
Rectal malignancies	11	17	14	42	0.52
Total number of patients	19	49	52	120	-
P-value	0.049	0.049	0.049	-	

In the normal frailty group, seven of the participants had colon cancer, one had stomach cancer, and 11 had rectal cancer. In the pre-frail group, 23 participants had been diagnosed with colon cancer, nine with gastric cancer, and 17 with rectal cancer. In the frail group, 21 patients had been diagnosed with colon cancer, 17 with gastric cancer, and 14 with rectal cancer. Colon and rectal malignancies were generally higher in the pre-frail group, while gastric malignancies were generally higher in the frail group. The relationship between the groups was statistically significant based on the Monte Carlo chi-square test (p = 0.049) (Table 2).

Thirty-two of the participants were readmitted to the hospital within 30 days. Of these, 13.7% were in the normal frailty group, 31.6% were in the pre-frail group, and 54.7% were in the frail group.

Wound healing and wound infections developed in 37 participants. These patients required longer hospitalizations due to the need for antibiotherapy. The mean hospitalization duration for this subgroup of participants was 13 days. Among the participants who developed wound site infections, four (10.8%) were in the normal frailty group, 13 (35.1%) were in the pre-frail group, and 20 (54%) were in the frail group.

The mean hospitalization duration was 5.1 days for participants in the normal frailty group, 6.5 days for those in the pre-frail group, and 8.4 days for those in the frail group. Hospitalization duration ranged from a minimum of one day to a maximum of 20 days, and the length of stay in the intensive care unit ranged from a minimum of one day to a maximum of 30 days.

## Discussion

Our knowledge of frailty in relation to the outcomes of patients exposed to surgery has expanded significantly in recent years, and it has been demonstrated in many studies that elderly patients with high FIS develop significant complications, experience loss of functional status, and have prolonged hospitalization after a wide range of elective or emergency surgical procedures [5-10].

This study demonstrates that patients with colon, rectum, and gastric malignancies who were part of the vulnerable group experienced prolonged hospitalization and/or ICU stays, and more nosocomial infections, including wound infections, compared to other groups. In a study of 260 emergency patients, Orouji JT et al. showed that frail patients have a higher rate of postoperative complications and mortality, and a longer hospital stay than non-frail patients (47% vs. 20%, p < 0.001).

There was no correlation between age and postoperative complications [11]. Makary MA et al. examined 594 elderly patients hospitalized for elective surgery and found that elderly and frail individuals are at an elevated risk of postoperative complications and longer hospitalizations [5]. The reason for not reaching this conclusion in our study may be both the presence of patients with heterogeneous malignancy diagnoses and the lower number of cases.

Albumin is still used as an indicator of nutritional status, even though there are many limitations to its efficacy in this respect. Amino acids, critical for wound healing following surgery, are transported by albumin, and adequate vascular oncotic pressure cannot be maintained in people with albumin deficiency, leading to edema in surgical wounds and impaired wound healing [12,13]. In this study, it was determined that mean albumin levels differed statistically significantly with age (p = 0.006); they are negatively correlated with FIS and age. As FIS increases, albumin blood plasma levels decrease. The postoperative wound infection rate for cancer patients was 54% higher than in other groups. Elderly people with a high FIS are more prone to wound infections.

In our study, we observed that FIS increased with age. Fried LP et al. report that although FIS were high in elderly adults and in patients with multiple diseases, the trend might be unrelated to comorbidities, advanced age, disability, or any specific disease [14]. Age seems to be an obstacle to oncological treatment appropriate for females with breast cancer. The majority of surgical procedures for breast cancer are now performed in an outpatient setting [15], and older female breast cancer patients are less likely to receive standard oncologic surgical treatment [16]. Several studies indicate that patients aged 85 years and older are still less likely to undergo surgery for breast cancer, even after controlling for comorbidities [17]. Patients older than 65 years with undifferentiated thyroid cancers have been shown to be less likely to receive treatment that adheres to the guidelines for thyroidectomy and lymph node dissection. Therefore, a preoperative evaluation of elderly patients requiring thyroidectomy is critical in deciding the appropriate treatment modality [18].

The most prevalent descriptions of frailty describe it as a syndrome characterized by progressive multiple-system regression, loss of physiological reserve, and increased vulnerability to illness and death. Some researchers define frailty in terms of physical parameters such as endurance, muscle strength, balance, weight loss, mobility, and physical activity [14,19,20], while other researchers perceive frailty in a larger sense that includes psychosocial factors and cognitive impairment [21-23]. Descriptions using physical deterioration as an indicator of frailty usually use instruments that measure performance and functional changes in mobility and strength.

We conducted this FI study based on a definition of frailty centered on the physical capacity of older people. Because our study participants are malignancy patients, their psychological status varies greatly.

We believe that our study provides valuable insights into the predictive capacity of the FI in elderly patients undergoing surgery for GIM and that the sample size of 120 elderly patients with a diagnosis of GIM alone has reasonable power. Preoperative calculation of the FI will be useful in minimizing postoperative complications, length of hospital stay and improving patient outcomes.

This study also has several limitations. First and foremost, the study is retrospective. The results of this study need to be supported by a prospective clinical trial. Secondly, this study covered a wide range of organs and procedures. Cases of colon, stomach, and rectal cancer were included together regardless of the surgical procedure performed. Some procedures and tumors may have been significantly more likely to carry postoperative risks than others, which may have confounded the results. Subgroup analyses would be useful in future research to address this issue. Our other limitation is our findings have limited generalizability due to the inclusion of single-center patients with only three GIM types (colon, stomach, rectal cancers). Therefore, multicenter studies are needed to confirm these findings. Another limitation of our study is the lack of long-term outcomes (e.g., survival rates, quality of life). Studies addressing long-term outcomes will reveal the lasting effects of FI.

## Conclusions

This study found that older patients with colon, rectal, and gastric cancers had longer hospital and ICU stays after surgery. These patients also experienced more postoperative complications, particularly surgical site infections. They were found to have high FIS. The most important conclusion of our study is that to minimize morbidity and mortality in elderly patients, preoperative frailty assessments should be performed routinely, and greater attention should be paid to these adverse conditions that may occur in these patients.
